# Predictors of Parental Coping During the Covid-19 Pandemic: A Survey in Germany

**DOI:** 10.3389/fpsyg.2021.715327

**Published:** 2021-09-10

**Authors:** Vera Clemens, Franziska Köhler-Dauner, Ute Ziegenhain, Jörg M. Fegert

**Affiliations:** Hospital of Child and Adolescent Psychiatry/Psychotherapy, University of Ulm, Ulm, Germany

**Keywords:** COVID-19, pandemic, adverse childhood experiences, parental coping, child maltreatment

## Abstract

The Covid-19 pandemic has been profoundly affecting nearly everybody, but families with minors have been hit particularly. Closure of schools and kindergartens, home schooling, and working from home have led to a profound upheaval in family life. Parental adverse childhood experiences (ACEs) are an important determinant for parenting behavior. Importantly, ACEs can increase the vulnerability to stress and impair coping strategies. The current pandemic leads to increased parental stress, a risk factor for harsh parenting behavior, Therefore, we aimed to assess the role of ACEs and sociodemographic factors associated to parental coping during the current pandemic. In a cross-sectional online survey, 687 parents of minors in Germany were included. Demographic and psychosocial factors associated to parental coping during the first lockdown due to the Covid-19 pandemic were assessed. Results show that younger age of the respective child, income loss, dissatisfaction with the sharing of childcare duties, and ACEs were significantly associated with an increase of potential harmful parenting behavior during the Covid-19 pandemic. An increase of dissatisfaction with the sharing of childcare duties during the pandemic was predicted by working from home and taking care of the children mainly by oneself, while sharing childcare duties with the partner equally resulted even in an increase of satisfaction with sharing of childcare duties during the pandemic. These findings demonstrate that a history of childhood adversity in a parent is a risk factor for harmful parenting during the pandemic. Parental satisfaction with sharing of caregiving is an important factor for parental coping during the pandemic. Sharing of caregiving between partners should be encouraged.

## Introduction

Since the beginning of the Covid-19 pandemic, life of families all over the world has changed unprecedentedly. An estimated 90% or more of children and adolescents globally have faced the effects of school closures (UNESCO, 2020). Social contacts have been limited, out-of-home leisure time activities have been canceled (Imperial College COVID-19 Response Team, 2020). Parents have had to support children with home schooling, while working from home in parallel. Financial pressure has risen in many families due to unemployment and wage cuts. Economic problems can lead to feelings of stress and consequent marital conflict (Elder, 1974; Elder and Conger, 2000). Altogether, this can severely affect parenting and – in the worst case – erupt in physical and psychological violence among families (Fegert et al., 2020a). First official data point toward an increase of child maltreatment during the pandemic (Nguyen, 2021; Salt et al., 2021).

The course of the current pandemic is hard to predict. Although, a first strict lockdown has ended in most parts of the world, several following pandemic waves have hit many countries and some even face or have seen new lockdowns. Therefore, it is important to identify predictors of families who struggle to master the situation and who consequently may need more support. The number of studies assessing parenting during the pandemic have been rising. They point toward high levels of stress (Marchetti et al., 2020; Romero et al., 2020; van Tilburg et al., 2020), anxiety, and financial burden (Fong and Iarocci, 2020) among parents and parenting-related exhaustion (Marchetti et al., 2020) during the pandemic. Importantly, higher levels of parental stress was shown to be associated with harsh parenting behavior and poorer parent child relationship since the beginning go of the pandemic (Chung et al., 2020; Romero et al., 2020).

One factor that may be of importance on how parents deal with pandemic-associated stressors are adverse childhood experiences (ACEs). ACEs include child maltreatment, in detail emotional, physical, and sexual abuse as well as emotional and physical neglect, and household dysfunction, comprising e.g., mental illness and substance abuse of any household member, violence against the mother, parental separation, and incarceration of a household member (Hughes et al., 2017). ACEs are frequent. In the German population, more than 40% have experienced at least one type of ACE, and nearly 9% have experienced four or more (Witt et al., 2019). Even in less stressful times, ACEs increase the risk for psychosocial and economic impairments as well as mental and somatic health problems in a dose-dependent manner (Felitti et al., 1998; Norman et al., 2012; Rehkopf et al., 2016; Clemens et al., 2018). Furthermore, ACEs can significantly impair attachment and relationships in later life (Thomson and Jaque, 2017), just as the relation to one’s own children. ACEs can significantly affect parenting (Bailey et al., 2012) and increase the risk for harmful parenting behavior including child maltreatment (Dixon et al., 2005a,b; DuMont et al., 2007; Bailey et al., 2012; Clemens et al., 2019). This so called cycle of violence was initially based on the research finding that physical abuse is an important predictor for violence in adulthood (Hunter and Kilstrom, 1979). It describes the intergenerational transmission of violent behavior – although, it is important to emphasize that most abused and neglected children do not turn violent themselves (Wright et al., 2019). However, experience of violence during childhood is the strongest predictor for the use of violence as disciplinary method for one’s own children (Witt et al., 2017).

The experience of adversity during childhood affects the long-term individual stress response (Bunea et al., 2017) and can increase vulnerability to stress and impair successful coping of stressful situations by increased emotional reactivity and decreased emotion regulation (Hein and Monk, 2017; Duffy et al., 2018). A purposeful response to a stressful or challenging life event such as a pandemic on the other hand can be understood as coping (Compas et al., 2017). The experience of ACEs during childhood is suggested to result in perceiving the environment as threatening and unpredictable, leaving no or little opportunity to impact or change the environment (Sheffler et al., 2019). Consequently, ACEs are associated with less successful coping strategies of stressful situations (Leitenberg et al., 2004). During the pandemic, the majority of parents feel stressed by social distancing and closure of schools and childcare facilities (Calvano et al., 2021), resulting in increased perceived parenting stress (Brown et al., 2020). Thus, a significant impact of ACEs on coping and parenting strategies during the pandemic can be assumed. Recently, in a smaller sample of mothers, we were able to demonstrate that maternal ACEs are associated with endangered parenting behavior during the current pandemic (Köhler-Dauner et al., 2021). To the best of our knowledge, however, besides, data on the role of parental ACEs are missing. Moreover, as ACEs are associated with other important predictors such as economical impairment, psychosocial well-being, and parenting, they may influence these other factors. Importantly, parental stress was already shown to be a risk factor for child maltreatment during the pandemic, such as job loss, and younger age of the parent and the child (Lawson et al., 2020; Calvano et al., 2021).

Here, we aimed to assess the role of ACEs and sociodemographic factors associated topotential harmful parenting actions and parental coping of the current pandemic. The hypothesis was that parents are at higher risk for less successful coping and harmful parenting behavior during the pandemic when they were exposed to ACEs themselves.

## Materials and Methods

### Study Design

We have conducted a cross-sectional online survey, which was available from May 18th to July 21th 2020. The first lockdown in Germany began on March 23, 2020 and ended *via* gradual relaxations – the first schools reopened on April 22, the openings of schools and kindergartens stretched to the end of June 2020. Inclusion criteria were age above 18years and informed consent. Information on the survey was distributed by our clinical homepage, social media, and print media and existing mailing lists from other studies and interested parties. In total, 1,399 participants have participated in the online survey. For the here presented analyses, only data of participants who stated to be currently parent of a minor were assessed (*N* =687).

### Ethics

Information on the study and data analysis were given, electronic informed consent was obtained from each participant. Participation was anonymous. Participants could withdraw from the survey at any moment without providing any justification. The study was conducted in accordance with the Declaration of Helsinki. After consultation with the ethics committee of the University of Ulm, due to the anonymous character of the survey, there was no requirement for an ethics vote.

### Measures

Socio-demographic questions included age, gender, educational level, and change of income during the pandemic and whether the participant has worked from home during the pandemic. In detail, the questions were “Did you work from home in the meantime during the start of the Covid-19 pandemic,” “Has the income available in your household fallen by more than a quarter since the start of the Covid-19 pandemic?”, “Who took care of the child during most of the time during the Covid-19 pandemic?”, “Please indicate on a scale of 0–100 how satisfied you are with the sharing of childcare duties between you and your partner (before the pandemic and now). The best conceivable satisfaction is marked with a ‘100,’ the worst with ‘0.’,” “To what extent do the following statements apply to you? Since the beginning of the Covid-19 pandemic…(1) stands for ‘does not apply at all,’ (7) for ‘applies very much’ – …I yell more at the child/…Am I more impatient with the child/…Am I increasingly afraid that my hand to smack the child/…Am I increasingly afraid that my partner will smack the child” and “Summarized, on a scale from 0 to 100…., (0) stands for ‘miserable,’ (100) stands for ‘excellent,’ – … how well have you mastered the challenges of the Covid-19 pandemic to date?/…how well the child has mastered the challenges of the Covid-19 pandemic/…how well the family has mastered the challenges of the Covid-19 pandemic.”

Regarding potential harmful parenting behavior, it was asked whether participants have yelled more at the child, whether they had been more impatient with the child, whether they had been more afraid to smack the child and whether they had been more afraid that their partner would smack the child since the beginning of the pandemic. Answers were possible on a Likert scale of 1–7, where (1) stood for “does not apply at all” and (7) for “applies very strongly.”

Moreover, the participants were asked about parental satisfaction with the sharing of childcare duties before and during the pandemic and their assessment of how they, their children, and their family had dealt with the challenges of the pandemic (all answers: Likert scale 0 for worst and 100 for best).

The ACEs were assessed using the German version of the ACEs Questionnaire, a standard tool for retrospective assessment of ACEs with satisfactory reliability (Cronbachs *α*=0.76; Wingenfeld et al., 2011). With the ACE Questionnaire, five forms of child maltreatment (physical abuse, emotional abuse, sexual abuse, physical neglect, and emotional neglect) and five forms of household dysfunctions (substance abuse and mental illness of a family member, intimate partner violence between parents, incarceration of a family member, and disappearance of a parent through divorce, death, or other reason) are assessed in a dichotomous manner (yes/no). A sum score of all types of experienced ACEs can be calculated (Felitti et al., 1998). No experiences of any ACE is the lowest score “0,” a score of “10” is the highest, meaning that a participant has experienced all 10 assessed forms of ACEs.

### Data Analyses

Statistical analyses were performed with SPSS version 21.

Linear regression analyses were performed in order to identify factors associated to parenting and successful coping of pandemic-related challenges. Sociodemographic variables, Covid-19 related variables (for parental satisfaction with the sharing of childcare duties: difference between parental satisfaction with the sharing of childcare duties values during and before the pandemic), and the number of experienced forms of ACEs were included into the model.

A two-way repeated measures ANOVA was used to test differences in parental satisfaction with the sharing of childcare duties before and during the pandemic (main effect time). Between groups with gender, working from home and organization of child care (main effect gender) and a differential effect of gender, working from home and organization of child care on parental satisfaction with the sharing of childcare duties over time was tested (interaction effect time×parental satisfaction with the sharing of childcare duties).

## Results

### Participants

Primary caregivers were predominantly female (*N* =615, 89.5%). The mean age of the sample was 41.4 (±7.4) years for females and 45.8 (±8.0) for males (age range f: 26–67, m: 33–71). The majority of the sample lived with a partner (f: *N* =518, 84.2%; m: *N* =65, 90.3%). Academic degree of participants was generally high with the majority holding a diploma from German secondary school qualifying for university admission (“Abitur”) or a university degree (f: *N* =416, 67.6%, m: *N* =56, 77.8%). A minority was affected financially by the pandemic, with a decreased income by more than a quarter (f: *N* =69, 11.2%, m: *N* =6, 8.3%).

Demographic information is displayed in Table 1.

**Table 1:** Sample characteristics.

	Female	Male	*p*
Number of participants	615 (89.5)	72 (10.5)	
Age			
Mean (SD)	41.4 (7.4)	45.8 (8.0)	<0.001
Age range	26–67	33–71	
Highest level of education			
University degree or diploma from secondary school	416 (67.6)	56 (77.8)	0.079
Other or no school diploma	199 (32.4)	16 (22.2)	
Decrease of income >25% since the pandemic	69 (11.2)	6 (8.3)	0.455
Working from home	279 (51.6)	37 (52.9)	0.839
Living with partner	518 (84.2)	65 (90.3)	0.175
Number of children in household			
1	164 (30.7)	22 (34.4)	
2	263 (49.3)	33 (51.6)	
3	93 (17.4)	7 (10.9)	
4	11 (2.1)	2 (3.1)	
5	3 (0.6)	0 (0.0)	0.663
Gender of the child who’s birthday is next			
Female	274 (50.6)	32 (49.2)	
Male	267 (49.4)	33 (50.8)	0.829
Mean age of the child who’s birthday is next	7.2 (4.6)	8.1 (5.2)	0.159
Satisfaction with sharing of childcare duties			
Before the pandemic (M, SD)	7.8 (2.4)	8.4 (2.2)	0.080
During the pandemic (M, SD)	7.3 (2.8)	7.7 (2.5)	0.286
Decreased during the pandemic	191 (38.8)	19 (30.6)	0.211
Childcare during the pandemic			
Mainly by oneself	313 (59.6)	6 (9.4)	
Mainly by partner	28 (5.3)	49 (45.3)	
Mainly equally by oneself and partner	126 (21.4)	24 (37.5)	
Mainly by someone else (School/Kindergarden/relatives, etc.)	58 (11.0)	5 (7.8)	<0.001
Number of ACEs (M, SD)	1.7 (1.9)	1.3 (1.4)	0.011

Presented as *N* (%) unless stated otherwise.

### Increase of Potential Harmful Parenting Behavior

**Figure 1:**
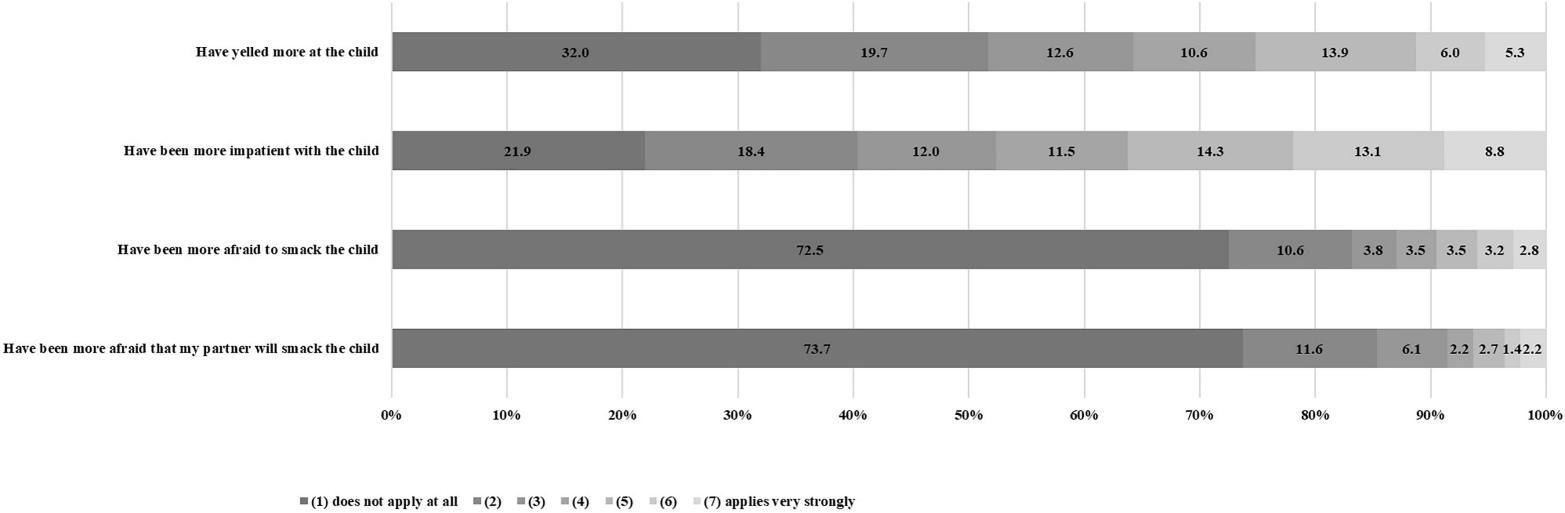
Increase of potential harmful parenting actions since the beginning of the pandemic. Change of potential harmful parenting behavior since the beginning of the pandemic.

First, we analyzed data on increase of potential harmful parenting behavior since the beginning of the pandemic. The majority of participants stated no or moderate increase of potential harmful parenting actions. However, the question whether they had yelled more at the child was answered by 5.3% of parents with “applies very strongly.” A total of 8.8% of parents reported they had been more inpatient with the child. For 2.8% of parents it applied very strongly that they had been more afraid to smack their child since the beginning of the pandemic, while for 2.2% it applied very strongly that they had been more afraid of their partner smacking their child (see Figure 1).

### Factors Associated to Parenting Behavior During the Pandemic

The results of the regression analyses revealed that a younger age of the child (*B*=−0.06, *p*<0.005), a decrease in parental satisfaction with the sharing of childcare duties compared to before the pandemic (*B*=−0.84, *p*<0.001) and decreased income by more than a quarter since the beginning of the pandemic (*B*=−0.51, *p*<0.05) were associated with more frequent yelling at the child.

Factors associated to higher scores for being more impatient with the child were younger age of the child (*B*=−0.08, *p*<0.001), decreased parental satisfaction with the sharing of childcare duties compared to before the pandemic (*B*=−0.09, *p*<0.001), and decreased income since the beginning of the pandemic (*B*=−0.91, *p*<0.001).

Affirmation of the sentence “I am increasingly afraid that I will smack the child” was higher if the respective child was younger (*B*=−0.04, *p*<0.05), if parental satisfaction with the sharing of childcare duties had decreased since the pandemic (*B*=−0.40, *p*<0.01) and higher numbers of ACEs were reported (*B*=0.13, *p*=0.001). Decreased income since the beginning of the pandemic lost significance after adding the number of ACEs into the analysis.

Concerns that the partner will smack the child were increased if participants reported a decrease of parental satisfaction with the sharing of childcare duties since the beginning of the pandemic (*B*=−0.25, *p*<0.05) and if a higher number of ACEs (*B*=0.15, *p*<0.001) were reported (for details see Table 2).

**Table 2:** Factors associated to potential harmful parenting actions during the first lockdown.

Since the beginning of the pandemic	Have yelled more at the child			Have been more impatient with the child			Have been more afraid to smack the child			Have been more afraid that my partner will smack the child		
	*B*	95% CI	*p*	*B*	95% CI	*p*	*B*	95% CI	*p*	*B*	95% CI	*p*
Gender	−0.316	−0.821; 0.189	0.219	−0.439	−0.968; 0.091	0.104	0.136	−0.297; 0.569	0.537	0.138	−0.236; 0.512	0.470
Age in years	−0.015	−0.049; 0.019	0.383	−0.003	−0.039; 0.033	0.865	0.003	−0.027; 0.032	0.863	−0.008	−0.033; 0.018	0.554
Living with a partner	0.258	−0.367; 0.884	0.418	0.164	−0.491; 0.819	0.623	0.356	−0.180; 0.892	0.192	0.171	−0.299; 0.640	0.475
Number of children living in household	0.113	−0.088; 0.315	0.268	0.062	−0.149; 0.273	0.564	0.080	−0.093; 0.252	0.366	0.102	−0.048; 0.252	0.183
Age of the respective child	−0.055	−0.099; −0.011	**0.015**	−0.084	−0.131;−0.038	**<0.001**	−0.038	−0.076; 0.000	**0.049**	−0.024	−0.057; 0.009	0.153
Satisfaction with sharing of childcare duties	−0.842	−1.161; −0.523	**<0.001**	−0.900	−1.235; −0.566	**<0.001**	−0.399	−0.672; −0.125	**0.004**	−0.254	−0.491; −0.016	**0.036**
Household income >25% less	−0.507	−0.994; −0.020	**0.042**	−0.911	−1.422; −0.400	**<0.001**	−0.387	−0.807; 0.034	0.072	−0.206	−0.573; 0.161	0.271
ACE total score	0.055	−0.031; 0.142	0.211	0.050	−0.041; 0.142	0.280	0.130	0.056; 0.205	**0.001**	0.148	0.083; 0.213	**<0.001**
*F* (df)	8.000 (8)			10.235 (8)			4.395 (8)			4.499 (8)		
*p* (total model)	<0.001			<0.001			<0.001			<0.001		
*R* ^2^	0.095			0.135			0.049			0.051		

*B*, unstandardized coefficient. Bold values are statistically significant (p < 0.05).

### Factors Associated to Change in Parental Satisfaction With the Sharing of Childcare Duties

A significant decrease in parental satisfaction with the sharing of childcare duties was seen in male and female participants (*F*=17.90, *p*<0.001). No significant difference regarding parental satisfaction with the sharing of childcare duties before and during the pandemic was seen for gender (*F*=0.24, *p*=0.623).

Working from home during the pandemic was associated with a lower parental satisfaction with the sharing of childcare duties (*F*=4.68, *p*<0.05). A stronger decrease of parental satisfaction with the sharing of childcare duties compared to before the pandemic was seen in participants who reported to have worked from home during the pandemic (*F*=4.39, *p*=<0.05).

**Figure 2:**
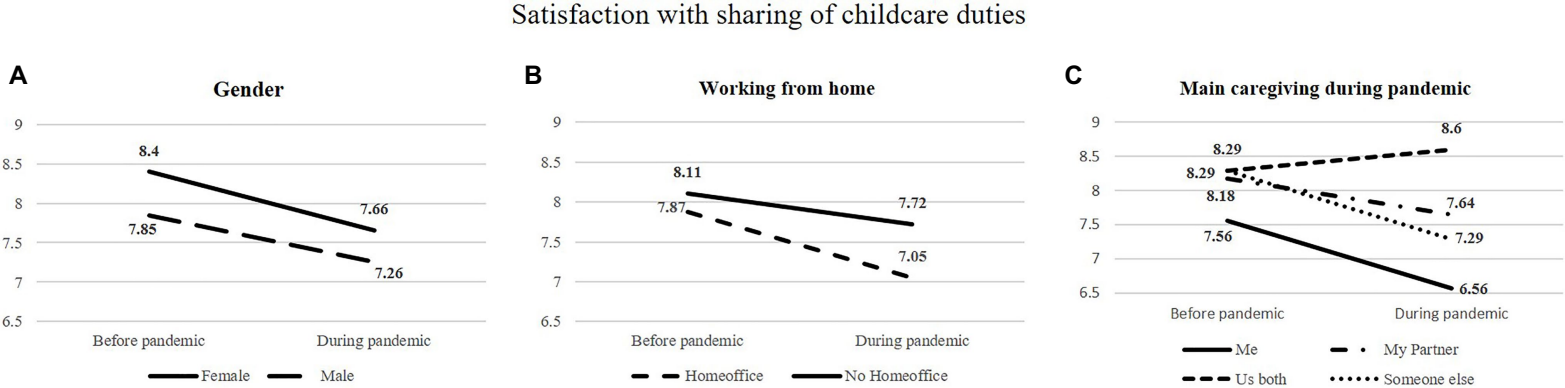
Satisfaction with the sharing childcare duties before and during the Covid-19 pandemic. Assessed *via* repeated measure statistics. **(A)** Satisfaction with the sharing of childcare duties in dependence of gender of the participants. While satisfaction with the sharing of childcare duties decreased for all participants during compared to before the pandemic, no difference was seen in regard to gender of the participants. **(B)** Satisfaction with the sharing of childcare duties in dependence of working from home. Working from home was associated with a lower satisfaction with the sharing of childcare duties and a stronger decrease during compared to before the pandemic (interaction significant). **(C)** Satisfaction with the sharing of childcare duties in dependence of who mainly has taken care for the children during lockdown. Satisfaction was significantly lower in participants who mainly have been taking care by themselves compared to participants who shared childcare equally.

While parental satisfaction with the sharing of childcare duties increased in participants who reported to share caregiving equally, parental satisfaction with the sharing of childcare duties decreased if participants reported that mainly themselves or mainly their partners provided childcare (*F*=20.56, *p*>0.001). Lowest grades of parental satisfaction with the sharing of childcare duties before and during the pandemic was reported by participants who reported to mainly provide care of the child by themselves (*F*=13.08, *p*<0.001; see Figure 2).

### Factors Associated to Coping With the Pandemic

Higher scores in affirmation of the question “How well have you mastered the challenges of the Covid-19 pandemic to date?” were reported if participants did not report decrease of income since the beginning of the pandemic (*B*=0.45, *p*<0.05) and parental satisfaction with the sharing of childcare duties had not decreased since the beginning of the pandemic (*B*=1.11, *p*<0.001).

Higher scores regarding the question how well the child mastered pandemic-associated challenges were associated to a higher academic degree of participants (*B*=0.34, *p*<0.05), an increase of parental satisfaction with the sharing of childcare duties since the beginning of the pandemic (*B*=0.84, *p*<0.001) and a lower number of parental ACEs (*B*=−0.17, *p*<0.001).

Families mastered the pandemic better if income (*B*=0.57, *p*<0.05) and parental satisfaction with the sharing of childcare duties had not decreased since the beginning of the pandemic (*B*=0.90, *p*<0.001, for details see Table 3).

**Table 3:** Factors associated to coping of the pandemic.

	How well oneself has mastered the challenges of the pandemic			How well the child has mastered the challenges of the pandemic			How well the family has mastered the challenges of the pandemic		
	*B*	95% CI	*p*	*B*	95% CI	*p*	*B*	95% CI	*p*
Gender	−0.090	−0.524; 0.344	0.685	−0.224	−0.689; 0.242	0.345	0.094	−0.341; 0.530	0.671
Age in years	−0.012	−0.044; 0.019	0.447	−0.011	−0.045; 0.022	0.506	−0.029	−0.060; 0.003	0.075
Living with a partner	−0.339	−0.888; 0.210	0.226	0.157	−0.433; 0.746	0.601	−0.024	−0.576; 0.527	0.931
Number of children living in household	−0.049	−0.233; 0.135	0.603	0.033	−0.168; 0.234	0.749	−0.016	−0.200; 0.169	0.868
Age of the respective child	0.027	−0.014; 0.068	0.200	−0.041	−0.085; 0.004	0.071	−0.001	−0.042; 0.040	0.960
Education	0.039	−0.267; 0.345	0.802	0.340	0.012; 0.668	**0.043**	0.091	−0.216; 0.398	0.559
Household income >25% less	0.449	0.003; 0.895	**0.048**	0.235	−0.243; 0.714	0.334	0.566	0.118; 1.014	**0.013**
Satisfaction with sharing of childcare duties	1.108	0.818; 1.398	**<0.001**	0.844	0.534; 1.155	**<0.001**	0.901	0.610; 1.191	**<0.001**
Working from home	0.151	−0.136; 0.437	0.302	0.069	−0.238; 0.376	0.660	0.131	−0.157; 0.418	0.371
ACE total score	−0.051	−0.129; 0.437	0.191	−0.172	−0.255; −0.088	**<0.001**	−0.058	−0.135; 0.020	0.144
*F* (df)	7.221 (10)			6.863 (10)			5.461 (10)		
*p*	<0.001			<0.001			<0.001		
*R* ^2^	0.136			0.130			0.106		

*B*, unstandardized coefficient. Bold values are statistically significant (p < 0.05).

## Discussion

To the best of our knowledge, this is the first study assessing the role of ACEs on paternal and maternal parenting behavior during the Covid-19 pandemic. Several factors critically predicted parenting behavior including “parental satisfaction with the sharing of childcare duties,” “decrease of income since the beginning of the pandemic” and “ACEs” as the most frequent ones. Importantly, the experience of adversity during childhood or adolescence was associated with an increased concern about physical violence against the child. This is crucial, as an increase of violence within the family was hypothesized for the pandemic due to increased stress and challenges in daily life such as home schooling and working from home at the same time, reduced support, decreased leisure time activities for stress reduction, financial pressure, and reduced social control (Fegert et al., 2020b). ACEs are known to significantly increase the risk for harmful parenting behavior, including physical maltreatment, known as the “cycle of violence” (Dixon et al., 2005a,b; DuMont et al., 2007; Bailey et al., 2012; Clemens et al., 2019). Subjects who have experienced ACEs are known to have a higher stress-vulnerability (Hein and Monk, 2017; Duffy et al., 2018). The current pandemic leads to increased levels of perceived parenting stress (Brown et al., 2020; Calvano et al., 2021). The here shown data underline that parents with ACEs are at higher risk for maltreatment and suggest that targeted prevention for parents with ACEs is needed – in particular during the current pandemic.

In a German sample of more than 1,000 parents, 30% reported an increase in children witnessing domestic violence during the pandemic, and more than 40% reported an increase in emotional abuse. Affected families were characterized by higher parental stress, job loss, and younger parent and child age (Calvano et al., 2021). In a sample of over 3,000 parents in the United States, job loss during the pandemic was associated with increased risk for child maltreatment (Lawson et al., 2020). In our sample, a decrease of household income since the beginning of the pandemic by more than a quarter was associated with increased fear of physical violence against and yelling at the child. Moreover, income decrease was associated with less well individual and familiar coping of the pandemic. Recession was proven to increase rates for all forms of child maltreatment in a wide variety of cultures (Huang et al., 2011; Schneider et al., 2017). Income loss may be associated with economic hardship and higher distress in parents – increasing pressure within families and the risk for physical and psychological violence. These results strongly underline the importance of economic support for families whose household income has fallen significantly due to the current pandemic.

Change of parental satisfaction with childcare duties was crucial for parenting behavior during the pandemic. A decrease of parental satisfaction with the sharing of childcare duties compared to before lockdown was associated with stronger concern about physical violence against the child, as withmore frequent yelling at the child, impatience and worse individual and familiar coping of the pandemic. Although for the latter ones of course a reciprocal association cannot be excluded, these results point toward the relevance of parental satisfaction with the sharing of childcare duties for parenting and mastering of the pandemic-associated challenges. Dissatisfaction with sharing of childcare duties may lead to increased parenting distress, which was shown to be associated to child abuse potential during the pandemic (Brown et al., 2020; Chung et al., 2020). In a recent study of over 800 mother-child dyads, connectedness to caregivers was an important predictor for child mental health (McArthur et al., 2021).

In our sample, parental satisfaction with the sharing of childcare duties decreased overall. In an Australian study assessing satisfaction with work-family balance and partners’ share, satisfaction decreased for most parents since the beginning of the pandemic (Craig and Churchill, 2020) – being in line with our results. However, in the study by Craig and Churchill (2020), before the pandemic, women were more dissatisfied than men, while during the pandemic, this difference narrowed. In our study, no significant difference was seen in regard to gender neither before nor during the pandemic. However, as the number of male participants was very low in our sample, the validity of this result may be limited.

While nearly 60% of female participants were the main caregiver for the children during lockdown, this was only the case for 9.4% of male participant. This finding is in line with other results from German studies, showing that during lockdown, females were the main caregivers for children (Zinn et al., 2020b).

Participants who worked from home during the pandemic had a significantly higher decrease in parental satisfaction with the sharing of childcare duties compared to other participants. This is easily understandable as parents of minors working from home had to guard the children and to support them in home schooling during lockdown – likely resulting in distress and overstrain. Consequently, having the main responsibility for childcare duties was associated with lower parental satisfaction with the sharing of childcare duties. Interestingly, in participants who shared childcare equally during the pandemic, satisfaction with the sharing of childcare duties increased. This is an encouraging finding. Despite the stress of the pandemic, taking these challenges together can even improve some aspects of family life.

Several limitations of our data have to be considered. Survey participants cannot be considered as representative for the general public, as data are based on a non-probability sample. Compared to data of a sample of parents derived from the Socio Economic Panel, a probability sample in Germany, assessed in a similar time frame, in our sample parents had a higher education, more children in the respective household were reported and rate of participants having a partner was higher (Zinn et al., 2020a). Therefore, the findings cannot be generalized for all families. As lower education correlates with higher rates of child maltreatment (Kotch et al., 1995; Berthold et al., 2019), the results of this study may tend to underestimate the negative impact of the pandemic on families. Accordingly, the results shown should be interpreted as an indication of possibly even greater stress in affected families. Questions on parenting before the pandemic are based on retrospective self-report, just as questions regarding ACEs, which may impair validity due to recall bias. There has been a critical debate on the validity of retrospectively assessed ACEs (Baldwin et al., 2019), but subjective reports of childhood maltreatment were shown to be highly relevant for adulthood (Danese and Widom, 2020). As to the best of our knowledge, no data on parenting during the Covid-19 pandemic are known yet from cohorts assessing ACEs and parenting prospectively, our findings are of high relevance.

In our cross-sectional study, causality cannot be deduced. Reciprocal associations – such as between parental satisfaction with the sharing of childcare duties and parenting behavior are likely. However, the presented results give an important insight into the relevance of factors such as economic pressure, parental satisfaction with the sharing of childcare duties, and ACEs on parenting during and coping with the pandemic. As the impact of economic pressure was already significant in our highly educated sample, it can be assumed that the impact of economic hardship may be even more relevant in a sample with a lower socioeconomic status, and consequently in a more representative sample.

Taken together, younger age of the child, economic pressure, dissatisfaction with the sharing of childcare duties, and ACEs are significantly associated with potential harmful parenting behavior during the Covid-19 pandemic. There is a need for targeted support for parents with ACEs and dissatisfaction with their family model regarding caregiving. Models where both parents share childcare duties shall equally be encouraged. Economic support is needed for families who have lost a significant part of their income due to the pandemic.

## Data Availability Statement

The raw data supporting the conclusions of this article was made available by the authors on reasonable request.

## Ethics Statement

Ethical review and approval was not required for the study on human participants in accordance with the local legislation and institutional requirements. The patients/participants provided their written informed consent to participate in this study.

## Author Contributions

VC interpreted the data and wrote the manuscript. FK-D and UZ supported recruitment of the sample. JF conceptualized the survey and supervised data analyses. All authors contributed to the article and approved the submitted version.

## Conflict of Interest

JF has received research funding from the EU, DFG (German Research Foundation), BMG (Federal Ministry of Health), BMBF (Federal Ministry of Education and Research), BMFSFJ (Federal Ministry of Family, Senior Citizens, Women and Youth), G-BA Innovationsfonds, several state ministries, State Foundation Baden-Württemberg, Volkswagen Foundation, Porticus Foundation, and Diocese of Rottenburg-Stuttgart. Moreover, JF received travel grants, honoraria, and sponsoring for conferences and medical educational purposes from APK, Deutschlandfunk, DFG, DJI, DKSB, Infectopharm, med update, UNICEF, several universities, professional associations, political foundations, and German federal and state ministries during the last 5years. JF holds no stocks of pharmaceutical companies.

The remaining authors declare that the research was conducted in the absence of any commercial or financial relationships that could be construed as a potential conflict of interest.

## Publisher’s Note

All claims expressed in this article are solely those of the authors and do not necessarily represent those of their affiliated organizations, or those of the publisher, the editors and the reviewers. Any product that may be evaluated in this article, or claim that may be made by its manufacturer, is not guaranteed or endorsed by the publisher.
